# Increased Expressions of IL-22 and Th22 cells in the coxsackievirus B3-Induced mice acute viral myocarditis

**DOI:** 10.1186/1743-422X-9-232

**Published:** 2012-10-11

**Authors:** Qing Kong, Weifeng Wu, Fan Yang, Yanli Liu, Yimin Xue, Mengsha Gao, Wenyin Lai, Xiaofen Pan, Yuluan Yan, Yu Pang, Yuanhua Deng

**Affiliations:** 1 Department of Cardiology, First Affiliated Hospital of Guangxi Medical University, Guangxi Cardiovascular Institute, Nanning, 530021, China

**Keywords:** Th22 cells, IL-22–IL-22R pathway, Acute viral myocarditis

## Abstract

**Background:**

Recently, a new subset of T helper (Th) cell that predominantly secret cytokine interleukin-22 (IL-22) is identified, termed Th22 cells. The Th22 subset has been demonstrated to be involved in immunity and tissue inflammation. However, the existence of Th22 cells and role of IL-22 in acute viral myocarditis (AVMC) remain unknown.

**Methods:**

BALB/c mice were intraperitoneally (i.p) infected with CVB3 for establishing AVMC models. Control mice were treated with phosphate-buffered saline (PBS) i.p. On day 14 post injection, frequencies of splenic Th22 cells were determined, productions of IL-22 and expressions of IL-22R (IL-22 receptor) were measured. To further investigate the effects of IL-22, AVMC mice treated with Anti-IL-22 neutralizing antibody were explored. The severity of AVMC were monitored; the frequencies of Th22 cells, the expressions of IL-22 and IL-22R were investigated; in addition to IFN-γ, inflammatory cytokines IL-17, TNF-α, IL-6 as well as IL-1β, were evaluated. Cardiac viral replication were detected.

**Results:**

Compared with control group, significant elevations of circulating Th22 cells and IL-22, cardiac protein and mRNA of IL-22, and IL-22R1 were demonstrated in AVMC group. Treatment of AVMC mice with Anti-IL-22 Ab exacerbated the severity of viral myocarditis, verified by lower survival rate, higher HW/BW ratios and cardiac pathological scores. Anti-IL-22 Ab decreased the frequencies of Th22 cells and the levels of IL-22, and increased the expressions of cardiac IL-22R1. Up-regulations of IL-17, IL-6 and TNF-α, down-regulations of IFN-γ proteins and gene expressions in the plasma and myocardium, were observed in Anti-IL-22 Ab group. Furthermore, neutralization of IL-22 significantly promoted cardiac viral replication.

**Conclusions:**

Our data indicate that the increased frequencies of IL-22-producing Th22 cells may play an important role in the pathogenesis of CVB3-induced mice AVMC, IL-22 may act as an myocardium-protective cytokine via the IL-22–IL-22R pathway, and suggest that targeting the Th22 cell and IL-22–IL-22R pathway could provide new therapeutic modalities for the treatment of CVB3-induced AVMC.

## Background

Acute viral myocarditis (AVMC) is characterized by myocardial inflammation and can progress to chronic dilated cardiomyopathy (DCM), a terminal condition requiring transplantation. Coxsackievirus B (CVB), an enterovirus of the Picornaviridae family, its infection is surprisingly prevalent, with an estimated 70% of the human population having exposed to these viruses. CVB3, a member of the CVB group, is the primary pathogen of viral myocarditis [1]. Accumulating data had indicated the dominant cause of myocardial cells damage in AVMC were inflammation and autoimmune responses triggered by the viral infection, mediated by T cells and their cytokines [1-3]. Emerging evidence have demonstrated that several Th subsets, such as Th1, Treg, Th17 but not Th9 cells, are involved in the pathogenesis of AVMC [4-8]. But the fundamental mechanisms responsible for AVMC are not completely clarified, and therapeutics for AVMC are restricted to supportive care including basic medications [9].

Recently, the Th22 cell has been recognized as a novel Th cell subset, which is characterized by abundant production of IL-22 but not IL-17 or IFN-γ [10-12]. This newly identified CD4^+^T cells clones have low or undetectable expression of transcription factor T-bet and RORγt (Th1 and Th17). With the CCR6^+^CCR4^+^CCR10^+^ phenotype, aryl hydrocarbon receptor (AHR) has been considered to be the key transcription factor of Th22 subset. Driven by the combined effects of IL-6 and TNF-a, naïve T cells differentiate toward the Th22 phenotype in human [10]. All of above evidence strongly support that the Th22 cell represent a terminally differentiated and independent T cells subtype. Demonstrated by emerging literature in kinds of diseases such as rheumatoid arthritis, atopic dermatitis, systemic sclerosis, human immunodeficiency virus (HIV) infection, Th22 cells were considered providing a unique contribution to tissue inflammation, virus infection and immune responses [13-16].

IL-22, the crucial Th22 signature cytokine, its role had been demonstrated by developing literatures, which reported that neutralization or genetic deletion of IL-22 could alleviate or exacerbate the severity of kinds diseases such as antigen-induced airway inflammation and T cell-mediated hepatitis [17,18], indicating that targeting IL-22 might represent a promising novel therapeutic approach for acute-phase response, immunity and inflammation. IL-22 mediates its effects via IL-22R, a heterodimeric transmembrane receptor complex consisting of IL-10R2 and tissue-specific IL-22R1 [19]. IL-10R2 is ubiquitously expressed, while the expression of IL-22R1 is restricted to nonhematopoietic cells. Signaling through IL-22R1 is restricted to IL-22, and tissues that lack IL-22R1 will not be a target for IL-22 under currently known molecular pathways. An increasing number of human-and mouse-based studies have indicated that IL-22–IL-22R interactions can modulate the expressions of considerable genes such as IL-17,IFN-γ,IL-1β,TNF-α,IL-6, and highlighted the fact that IL-22–IL-22R interactions are an integral pathway through which cells of the innate and adaptive immune responses regulate immunity and inflammation [20-22].

The existence of Th22 cells in CVB3-induced mice AVMC is still unclear. The roles of both IL-22 and the IL-22–IL-22R pathway in the pathogenesis of AVMC remain to be clarified. Therefore, our present study examined the frequencies of Th22 cells, investigated the expressions of IL-22 and IL-22R, targeted of IL-22 in vivo in mice AVMC with the use of neutralizing Anti-IL-22 antibodies, and aimed to preliminary explore the presence of Th22 cells and functional characteristics of IL-22 in AVMC.

## Results

### Evaluation for the severity of AVMC

**Figure 1:**
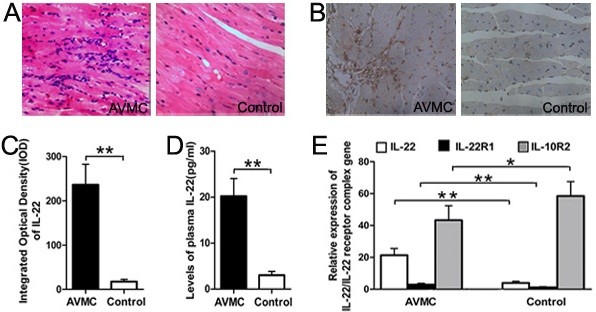
**Elevated expressions of IL-22 and IL-22R in AVMC, on day 14 post infection.A**. Representative of myocardial histopathologic images in AVMC and control groups (H&E, original magnification × 400). **B**. Representative of IL-22 immunohistochemistry images in heart tissue (Dark brown granules, original magnification × 400). **C**. Morphometric quantitation of cardiac IL-22 protein expressions. **D**. ELISA analysis of IL-22 protein levels in the plasma. E. Relative cardiac expressions of IL-22–IL-22 receptor complex gene were detected by RT-PCR. *p < 0.05, **p < 0.01. Data are mean ± SD. AVMC, Acute Viral Myocarditis.

The signs of AVMC were apparent in the AVMC group, including weakness, coat ruffling, irritability, back arching, lethargy, anorexia and weight loss. The cardiac pathological scores of AVMC were dramatically increased on day 14 post infection (2.6 ± 0.4). In contrast, the pathological scores of heart sections in the control group was 0, as neither inflammatory cell infiltration or necrosis lesion was observed (Figure 1A, *p*<0.05). On day 14 post injection, 5 of 20 mice died in the AVMC group. Specifically, on day 7,8,10,11 post infection, there were 1,1,2,1 mice dead in the AVMC group. Contrary, 0 of 12 mice died in the control group. Statistical differences were observed when comparing the survive rate of AVMC with control (*p*<0.05).

### Elevated expressions of IL-22 and IL-22R in AVMC

On day 14 post infection, compared with those in control mice treated with PBS only, much higher levels of the IL-22 protein and gene transcripts were detected in myocardium in AVMC mice, respectively (236.24 ± 43.26 vs. 17.45 ± 5.21, 21.28 ± 4.28 vs. 4.28 ± 0.97, both *p*<0.01, Figure 1B,C and E). As shown in Figure 1D, the significantly increased level of the circulating IL-22 protein in the AVMC group was observed compared with that in the control group (20.21 ± 3.81 vs. 3.02 ± 0.83pg/ml, *p*<0.01). Meanwhile, the relative cardiac gene expression of IL-22R1, the specific receptor of IL-22, was obviously up-regulated in the AVMC group, compared with that in the control group (3.01 ± 0.78 vs. 1.05 ± 0.29, *p*<0.01). In contrast, the relative cardiac transcripts of IL-10R2 in the AVMC group were less than those in the control group (43.28 ± 9.14 vs. 58.5 ± 9.01, *p*<0.05).

### Increased proportions of Th22, Th17 and Th1 cells in AVMC

**Figure 2:**
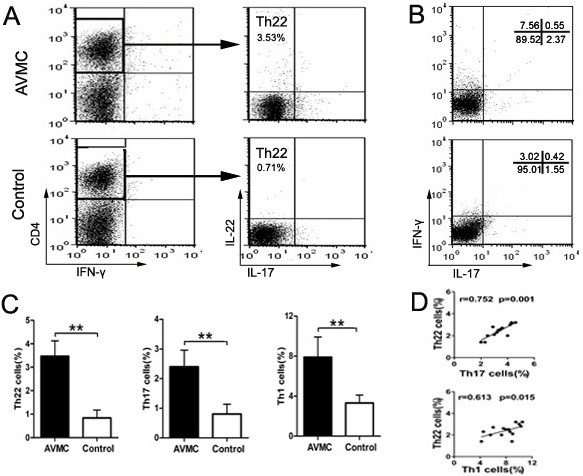
**Increased proportions of Th22, Th17 and Th1 cells in AVMC were investigated by flow cytometry analysis, on day 14 post injection.A**.Th22 cells within CD4^+^T cells were identified based on their expressions of IL-22^+^IL-17^-^IFN-γ^-^CD4^+^. **B**.Th17 and Th1 cells within CD4^+^T cells were determined based on their expressions of IL17^+^CD4^+^ and IFN-γ^+^CD4^+^. **C**. Comparisons of Th22, Th17 and Th1 cells in AVMC and control groups. **D**. The correlation analysis of Th22 and Th17, Th22 and Th1 cells in AVMC group. Each point represented an individual mouse. **p < 0.01. Data are mean ± SD.

Flow cytometry experiments were performed on mononuclear cells from spleen with gating on CD4^+^, on day 14 post injection. We detected Th22, Th17 and Th1 cells in both AVMC and control groups (Figure 2A,B). As shown in Figure 2C, the percentage of the pure Th22 cell (IL-22^+^IL-17^-^IFN-γ^-^CD4^+^) represented a higher value in the AVMC group (3.47 ± 0.66%), showing a significant increase in comparison with that in the control group (0.84 ± 0.33%, *p*<0.01). Meanwhile, the percentage of Th17 cells in the AVMC group was distinctly higher than that in the control group (2.41 ± 0.55% vs. 0.81 ± 0.33%, *p*<0.01). Similarly, the significantly increased level of the Th1 cell was observed in AVMC (7.89 ± 2.01%) compared with that in the control group (3.32 ± 0.79%, *p*<0.01, Figure 2C). It was established that the numbers of Th22 cells in the AVMC group were correlated positively with the numbers of Th17 and Th1 cells (both *p*<0.05, Figure 2D).

### Neutralization of IL-22 exacerbated the severity of AVMC

**Figure 3:**
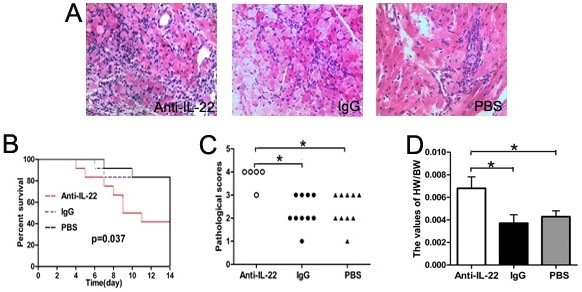
**Neutralization of IL-22 exacerbated the severity of AVMC on day 14 after infection.A**. Representative of myocardial histopathologic images in Anti-IL-22 Ab,IgG control and PBS groups (H&E, original magnification × 400). **B**. Survival rate was significantly decreased in Anti-IL-22 Ab group. **C**. The pathological scores in different groups. Each point represented an individual mouse. **D**. The ratios of HW/BW in different groups. *p< 0.05.

As shown in Figure 3, the results showed that Anti-IL-22Ab accelerated the development of myocarditis and exacerbated the severity. Firstly, the number of mice surviving to 14 day was 5, 10 and 10 for Anti-IL-22 Ab, IgG control and PBS groups, respectively. The survival rate of the Anti-IL-22 group was significantly decreased compared with those in IgG control and PBS groups (Figure 3B, both *p*<0.05). Consistently, both the pathological scores of heart sections and the values of HW/BW in mice receiving Anti-IL-22Ab were higher than those in PBS and IgG control groups (Figure 3C-D, all *p*<0.05), on day 14 post infection. Regarding the survival rates, ratios of HW/BW and cardiac pathological scores, no significant differences were observed between PBS and IgG control groups (all *p*>0.05).

### Neutralization of IL-22 decreased the percentage of the Th22 cell and dysregulated the expressions of the IL-22–IL-22R pathway

**Figure 4:**
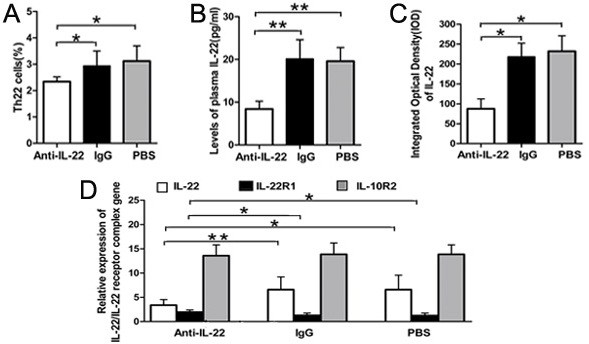
**Neutralization of IL-22 decreased the percentage of the Th22 cell, dysregulated the expressions of IL-22–IL-22R pathway on day 14 post infection.A**. The result of statistical analysis for the alterations of Th22 cells in Anti-IL-22 Ab,IgG control and PBS groups, investigated by flow cytometry. **B**. The levels of plasma IL-22 were measured by ELISA. **C**. Morphometric quantitation of cardiac IL-22 protein expressions were explored by IHC. **D**. Relative cardiac expressions of IL-22/IL-22 receptor complex gene were detected by RT-PCR. *p < 0.05, **p < 0.01. Data are mean ± SD.

As shown in Figure 4A, the alterations of Th22 cells in Anti-IL-22 Ab, IgG control and PBS groups were investigated by flow cytometry, on day 14 post infection. The percentage of Th22 cells in the Anti-IL-22 Ab group was less than those in IgG control and PBS groups (both *p*<0.05). No significant difference was observed between PBS and IgG control groups (*p*>0.05). The effects of Anti-IL-22 Ab on the expressions of the IL-22–IL-22R pathway were further addressed. Compared with those in IgG control and PBS groups, marked reduction of the circulating level of the IL-22 protein was detected in the Anti-IL-22 Ab group (Figure 4B, both *p*<0.01). As shown in Figure 4C and D, higher levels of the IL-22 protein and gene expression in myocardium lesions were observed in the Anti-IL-22 Ab group (all *p*<0.05). Regarding the levels of the IL-22 protein and transcripts, no significant differences were observed between PBS and IgG control groups (all *p*>0.05). At the same time, compared with those in PBS and IgG control groups, the level of cardiac IL-22R1 mRNA was increased significantly after IL-22 neutralization (both *p*<0.05, Figure 4D). The level of IL-22R1 did not exhibit distinct difference between IgG control and PBS groups. In contrast, IL-10R2 gene expressions were comparable among the Anti-IL-22 Ab, IgG control and PBS groups (all *p*>0.05).

### Neutralization of IL-22 increased the productions of IL-17, TNF-α and IL-6, decreased the level of IFN-γ

**Figure 5:**
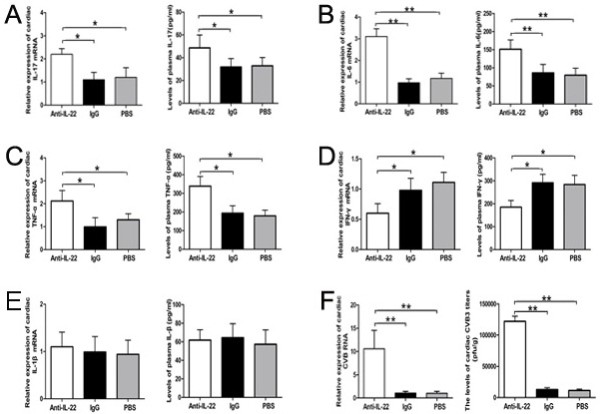
**Neutralization of IL-22 increased the productions of IL-17, TNF-α and IL-6, decreased the level of IFN-γ, promoted cardiac viral replication on day 14 post infection.** Relative cardiac expressions of IL-17, TNF-α, IL-6, IFN-γ and IL-1β were investigated by RT-PCR, and the levels of their circulating protein were measured by ELISA in Anti-IL-22 Ab, IgG control and PBS groups. **A-E:** IL-17, IL-6, TNF-α, IFN-γ and IL-1β, respectivity. **F**. The cardiac CVB3 titers and relative expressions of CVB3 RNA were investigated by PFU and RT-PCR. *p < 0.05, **p < 0.01. Data are mean ± SD.

On day 14 post injection, the cardiac mRNA expressions of inflammation cytokines such as TNF-α, IL-17A, IL-6, IL-1β, as well as IFN-γ were detected by RT-PCR. Moreover, their protein productions in plasma were measured by ELISA. As shown in Figure 5A-C, compared with those in PBS and IgG control groups, the levels of IL-17A,TNF-α and IL-6 mRNA and proteins were increased dramatically in the Anti-IL-22 Ab group (*p*<0.01 or *p*<0.05). These levels did not show significant differences between PBS and IgG control groups (all *p*>0.05). However, the expressions of the IFN-γ mRNA and protein in the Anti-IL-22 Ab group were lower than those in PBS and IgG control groups (Figure 5D, all *p*<0.05), and no significant difference was detected between PBS and IgG control groups (*p*>0.05). No significant changes in the levels of the IL-1β transcripts and protein were observed among Anti-IL-22 Ab, PBS and IgG control groups (Figure 5E, all *p*>0.05).

### Neutralization of IL-22 promoted viral replication

Compared with those in PBS and IgG control groups, the levels of cardiac CVB3 RNA and CVB3 titers in the Anti-IL-22 Ab group were elevated markedly, indicated by RT-PCR and plaque-forming unit (PFU) experiments (Figure 5F, all *p*<0.01). However, no obvious differences in these levels were observed between PBS and IgG control groups (*p*>0.05).

## Discussion

There is growing evidence that T cell activation and its cytokine expression play a key role in the pathogenesis of CVB3-induced AVMC, such as IFN-γ-producing Th1 cells and IL-17-producing Th17 cells [4-7]. Recently, several works related to Th22 cells have broadened our understanding of T cell-mediated tissue inflammation and immunity [13-16]. Pure Th22 cells represents a distinct CD4^+^T cell subset characterizing by the secretion of IL-22, but not IL-17 or IFN-γ, thus is identified as IL-22^+^IL-17^-^IFN-γ^-^CD4^+^T cells [10-12,16]. IL-22, the crucial cytokine of Th22 cells, mediates its effects via IL-22R and propagates downstream signals including JAK/STAT, ERK, JNK, PI-3K and p38 MAP kinase pathways [19,23,24]. A considerable amount of data indicated that the Th22 cell and its crucial cytokine IL-22 could exert either pro-inflammatory or anti-inflammatory activities depending on the context [13-18,22]. In the present work, we tried to exploit the potential that Th22 cells may participate in the inflammatory and immune response in AVMC. As excepted, higher percentage of the Th22 cell was observed in CVB3-induced mice AVMC, similarly to those of Th17 and Th1 cells. Our data uncovered the evidence of significant elevations in circulating Th22 cells and IL-22 proteins, cardiac mRNA and protein expressions of IL-22 in AVMC. It seems that infective and immunity microenvironment of AVMC is suitable for the differentiation and proliferation of Th22 cells. These novel findings confirm an individual signature for the IL-22-producing Th22 subset in AVMC, and provide a cellular target for therapeutic intervention and may also shed light on thus far unknown pathways in the control of AVMC.

Based on the observation that levels of IL-22 and IL-22 receptor were elevated in AVMC, we examined the efficacy of IL-22 in the AVMC mouse model, with the use of anti-IL-22 antibody (Anti-IL-22Ab) [20,25]. Functional studies in human or murine model systems have indicated that IL-22 could be either pathologic or protective, depending on the context in which it was expressed [17,18,22,26,27]. Our results proved that neutralization of IL-22 exacerbated the severity of AVMC, which was verified by the lower survive rate, higher values of HW/BW and pathological scores of heart sections. Meanwhile, down-regulations of circulating Th22 cells and IL-22 were observed in the Anti-IL-22Ab group. The Th22 cell is not the unique source of IL-22. However, Both the pathogenic effect of Anti-IL-22 Ab in our AVMC model and protective role of the IL-22-Ig fusion gene in experimental autoimmune myocarditis (EAM) model [28], suggest that this cytokine has an important myocardium-protective role regardless of the cell source.

Emerging literature indicate that IL-22 participates in immunity and tissue inflammation through the IL-22–IL-22R pathway [20,22]. To explore the role of this pathway in AVMC, we firstly investigated the expressions of cardiac IL-22 and IL-22R, the latter which consists of IL-22R1 and IL-10R2. Because signaling through IL-22R1 is restricted to IL-22, we focused on IL-22 and IL-22R1 [22]. Compared with IL-22 and IL-22R1, dramatically higher relative cardiac expression of IL-10R2 was detected in mice, consistence with previous data that IL-10R2 was highly expressed [20,22]. Compared with those in the control group, significant elevations of IL-22 and IL-22R1 were identified in the AVMC group. Although the major IL-22-producer are immune cells, it has been confirmed that IL-22R1 expression is absent on immune cells but instead restricted to tissues, thus IL-22 acts as a middle-men between the immune system and its environment [22]. These increases of cardiac IL-22 and IL-22R1 may strengthen signaling directionality from the immune system to myocardium, and are thus involved with the pathogenesis of AVMC. After IL-22 was blockade with a neutralizing antibody in AVMC, down-regulations of IL-22 and up-regulations of IL-22R1 were observed. These data are consistent with a previous study that IL-22R1 expression was decreased after treatment with rIL-22 but increased after IL-22 neutralization, while IL-10R2 was not changed significantly in liver ischemia-reperfusion injury model, by RT-PCR analysis [20]. These phenomena may be explained by the following mechanism: IL-22R1 has been recognized as a driver of inflammation, and has a autoregulatory feedback loop that amplifies the level of IL-22 [29,30]. When IL-22 is decreased by neutralization of anti-IL-22 Ab, IL–22R1 may be increased by autoregulatory feedback loop that promotes the levels of IL-22. And the promotion of IL-22R1 further aggravates the myocarditis in the anti-IL-22 group. On the other hand, IL-10R2 is constitutively expressed on virtually all cells types at high levels and is an component of the receptor complexes for IL-22, IL-10, IL-26, IL-28, and IL-29, thus the effect of anti-IL-22 on the expression of IL-10R2 is not significant. Further investigation with the use of heart-specific IL-22 transgenic mice and mice that lack IL-22R1 will be of particular interest to clarify the dysregulation of the IL-22–IL-22R pathway in AVMC.

Our data suggest that dysregulated IL-22–IL-22R signal can modulate expressions of considerable genes and regulate the productions of inflammatory cytokines, verified by the fact that IL-22 neutralization significantly promoted the circulating and cardiac IL-17, IL-6, TNF-α, which have been identified as important pro-inflammatory cytokines and exacerbating myocarditis by inflammation and immunity [4,5]. More specifically, dysregulated IL-22–IL-22R pathway in the Anti-IL-22 Ab group contributed to cardiac viral replication, and decreased the level of IFN-γ, which was considered as the vital defenders against viral infection [28,31]. Our data was consistent with the previous observation that IL-22 contributed to resistance to viral infection [32], highlighting a role for IL-22-producing Th22 cells in controlling cardiac viral replication in AVMC. It seems whether IL-22 response modulates the expression of IL-1β is context dependent because no significant change of IL-1β production was observed in the Anti-IL-22 Ab group [20,33,34].

To our knowledge, this is the first demonstration that Th22 and IL-22–IL-22R pathway may participate in the development of AVMC but with several limitations. First, the neutralization of IL-22 antibody biological activity was not fully quantified, and we have not addressed the mechanism responsible for proliferation of Th22 cells in mice. Whether the decreased Th22 cells in the anti-IL-22Ab group was due to the ab neutralizing or impaired proliferation, required future study. Second, Th22 has been regarded as the most important IL-22-producing CD4^+^T cells (71.9%) [16], elucidation of the role of the IL-22–IL-22R pathway would be helpful to understand the whole picture of Th22 cells-involved in AVMC. However, the source of IL-22 was not limited by the Th22 cell. NKT,Th17, Tc17, innate-like γδT, even in Rag2^−/−^ mice which lack of T cells, are IL-22 producers in mice [22,35]. Thus the myocardium-protective role of IL-22 could not be used to absolutely identify the Th22 function. In future studies on transcription factor AHR, chemokine receptor CCR6, PI-3 kinase pathway based-screening, will be of benefit to understanding of the Th22 cell regulation during inflammatory responses in AVMC [23,24]. Furthermore, whether RORC reflects the capacity to produce IL-17 and IL-22 in AVMC, is an interesting open issue.

## Conclusions

In summary, our preliminary data demonstrate that enriched IL-22-producing Th22 cells represent a distinct T cell subset in CVB3-induced mice AVMC. IL-22 may play a myocardium-protective role through the IL-22–IL-22R pathway in AVMC. Further studies focusing on the differentiation, regulation, downstream pathways of IL-22-producing Th22 cells, especially in human systems, are required to comprehensively explore the therapeutic potential of Th22 cells in AVMC.

## Methods

### Mice

Specific pathogen-free male BALB/c mice aged 6 week were purchased from the Hunan Laboratory Animal Centre, Chinese Academy of Sciences, Hunan, China (Certificate No. SCXK (Xiang) 2009–0004). All animals were kept in the pathogen-free mouse room in the experimental animal center (Experimental Animal Center of the Guangxi Medical University, Nanning,China), and all experiments were carried out in accordance with protocols approved by Guangxi Medical University Animal Ethics Committee.

### Virus

The CVB3 (Nancy strain, from Institute of immunology of Guangxi Medical University) was maintained by passage through Hep-2 cells. The virus titer determined by PFU assay was 1×10^8^. The CVB3 was diluted in PBS (Solarbio Science & Technology Co, Ltd, Beijing, China). BALB/c mice were infected by an intraperitoneally injection (i.p) of 100 μl PBS containing approximately ∼10^6^ PFUs of the virus for establishing AVMC models.

### Induction of AVMC

A total of 32 mice were randomly divided into two groups: 1) in the AVMC group, BALB/c mice were treated with CVB3 (100 μl per mouse) i.p. (n = 20); 2) mice administered i.p. with PBS were taken as control (n = 12). The day when mice were injected i.p. was defined as day 0. All surviving animals were sacrificed on day 14 after infection. Hearts and spleens were removed aseptically as fresh specimens to be measured, blood was harvested and the plasma was prepared for study.

### Neutralization of IL-22

For in vivo IL-22 neutralization, a total of 36 mice which infected with CVB3 (AVMC mice) were randomly divided into three groups: administered either Anti-IL-22 Ab (50 μg per mouse; R&D Systems, Inc. Minneapolis, MN.AF582; n=12,Anti-IL-22Ab group); an normal IgG control (50 μg per mouse; R&D Systems,Inc. Minneapolis,MN.AB-108-C; n=12, IgG control group); or PBS (n=12, 50 μg per mouse; PBS group) i.p., at day 0. All surviving animals were sacrificed on day 14 after CVB3 infection. The survival rates and values of the heart weight/body weight (HW/BW) were recorded. The hearts, spleens and plasma were collected.

### Histopathology

The ventricular tissues of the hearts were fixed in 10% formalin, then embedded in paraffin. After sectioned along the entire length of the heart into 5-μm sections, and stained with H&E (hematoxylin and eosin), histopathological change was observed by using light microscopy (Nikon Eclipse E800 Microscope, Kawasaki, Kanagawa, Japan). Pathological scores were graded by two independent pathologists separately in a blinded manner based on the following semi-quantitative scale: 0, no inflammatory infiltrates; 1, small foci of inflammatory cells between myocytes or inflammatory cells surrounding individual myocytes; 2, larger foci of 100 inflammatory cells or involving at least 30 myocytes; 3, 10% of a myocardial cross-section involved; 4, 30% of a myocardial cross-section involved [3].

### Flow cytometry

Spleens from mice were collected aseptically. After mincing, splenic cells were gently dispersed through nylon mesh into a single-cell suspension. Then these splenic mononuclear cell suspension was washed with RPMI 1640 (Gibco, USA). The lymphocyte fractions of these samples were obtained by Ficoll-Plaque (Solarbio Science & Technology, China) gradient centrifugation. Then cells were resuspended in RPMI 1640 medium with 10% FCS (Gibco, USA), stimulated with phorbol myristate acetate (PMA, 25ng/ml, Sigma- Aldrich, USA) and ionomycin (1μg/ml, Sigma-Aldrich) in the presence of GolgiPlug(1 ul/10^6^cells, BD Biosciences) at 37°C, 5% CO_2_ of a 24-well culture plate. After 4h incubation, the cells were harvested and stained with phycoerythrin Cye-5- conjugated anti-mouse CD4 (PE-Cye-CD4, BD Biosciences). Then cells were stained intracellularly with anti–IL-22, –IL-17, or –IFN-γ mAb conjugated with APC, PE, or AF488(Alexa-Fluor®488)after fixation and permeabilization (BD Biosciences, eBioscience), analyzed on a FACS-Calibur flow cytometer (BD Bioscience). Cell Quest soft-ware (BD Biosciences) was used for data acquisition. Pure Th22 cells were defined as IL-22^+^IL-17^-^IFN-γ^-^CD4^+^.

### Immunohistochemistry

Immunohistochemical staining was performed by the streptavidin-biotin complex method according to the manufacturer’s instructions. Rabbit polyclonal antibodies against mouse IL-22 (Abcam, USA) at a 1:200 dilution were used as primary antibodies. The sections were washed and stained using the streptavidin-biotin complex kit (Boster, Wuhan, China). After the sections were rehydrated, endogenous peroxidase activity was blocked with 3% hydrogen peroxide for 10 min at room temperature. Then the sections were first incubated in 5% bovine serum albumin for 20 min and then in the primary antibody for 24h at 4°C, and incubated with the streptavidin-biotin complex for 20 min. At last, the sections were developed with 3,3-diaminobenzidine (Boster, Wuhan, China) and observed under a light microscope. Instead of primary antibody, non-immune goat serum was taken as a control. IL-22 deposition in the cytoplasm and cytomembrane of myocardium were evaluated semi-quantitatively according to the extent and intensity of staining using Image-Pro Plus Version 6.0 (Media Cybernetics, Bethesda, MD). 5 fields from each slice were randomly selected by two pathology experts, who were unaware of the groups and evaluated by integrated optical density (IOD).

### Plaque-forming assay

Viral titers were determined by standard plaque formation assay. After part of the heart was weighed and homogenized in 2 ml PBS, three freeze-thaw cycles and centrifuging at 2000 rpm for 10 min were undertaken, then supernatant was sequential 10-diluted in RPMI 1640 medium. The HeLa cell monolayers were incubated with the supernatant for 1 h at 37°C, 5% CO2, in six-well plates, washed in PBS, and covered with 2 ml 0.4%agar, DMEM, and 5%FCS. After 72 h of cultivation, the monolayers were fixed in para formaldehyde and stained in crystal violet, and the numbers of plaques were counted. Viral titers were expressed per organ weight (in grams).

### Real-time RT-PCR

The total RNA of homogenized heart tissues was extracted with TRIZOL Reagent® (Invitrogen,USA), and then reverse transcripted into cDNA with an Reverse Transcription kit (Ferma, CA) according to the manufacturer’s instructions. Primers for IL-22,IL-22R1,IL-10R2,IL-17A,IFN-γ,IL-1β,TNF-α,IL-6, CVB3,and the housekeeping gene β-actin are designed by Primer Premier 5.0 (Table 1). Real time-polymerase chain reaction (RT-PCR) was performed using an ABI 7500 Sequence Detection System (Applied Biosystems, Foster City, CA) using SYBR green. After an initial denaturation step for 3 min at 94°C, a three-step cycling procedure (denaturation at 94°C for 30 sec, annealing at 60°C for 30 sec, and extension at 72°C for 60 sec) was used for 35 cycles. The relative gene expressions were normalized to the level of β-actin transcripts and quantified by the ▵▵C_T_ method using 7500 System Sequence Detection software (Applied Biosystems). All reactions were performed in at least duplicate for each sample.

**Table 1:** Sequences of primers for real-time RT-PCR

**Molecule**	**Sequence (5′~3′)**
TNF-α	sense: AGTCCGGGCAGGTCTACTTT
[GenBank:21926]	anti-sense: TTGGACCCTGAGCCATAATC
IL-6	sense: ACAGAAGGAGTGGCTAAGGACC
[GenBank:16193]	anti-sense: TAGGCATAACGCACTAGGTTT
IL-22	sense: CGATTGGGGAACTGGACCTG
[GenBank:50929]	anti-sense: GGACGTTAGCTTCTCACTTT
IL-22R1	sense: CTACGTGTGCCGAGTGAAGA
[GenBank:230828]	anti-sense: AAGCGTAGGGGTTGAAAGGT
IL-10R2	sense: GCCAGCTCTAGGAATGATTC
[GenBank:16155]	anti-sense: AATGTTCTTCAAGGTCCAC
CVB3	sense: CGGTACCTTTGTGCGCCTGT
[GenBank:JQ040513.1]	anti-sense: CAGGCCGCCAACGCAGCC
IFN-γ	sense: CTCAAGTGGCATAGATGTGGAAG
[GenBank:15978]	anti-sense: GCTGGACCTGTGGGTTGTTGA
IL-1β	sense: CAGGATGAGGACATGAGCACC
[GenBank:16176]	anti-sense: CTCTGCAGACTCAAACTCCAC
IL-17A	sense: GTGTCTCTGATGCTGTTG
[GenBank:16171]	anti-sense: AACGGTTGAGGTAGTCTG
β-actin	sense: AATTCCATCATGAAGTGTGA
[GenBank:11461]	anti-sense: ACTCCTGCTTGCTGATCCAC

### Cytokine assay

Cytokine content was determined by enzyme-linked immunosorbent assays on plasma. The amounts of IL-22 were detected using the Quantikine Mouse IL-22 Immunoassay (Biolegend,Cat.No.436307,USA). The levels of IL-17A,IFN-γ, IL-1β,TNF-α,IL-6 in mice were determined by ELISA kits (Shanghai ExCell Biology Inc. China), according to the manufacturer’s instructions. The sensitivity of ELISA kits for IL-22,IL-17,IFN-γ,IL-1β,TNF-α,IL-6 was 5,7,7,7,4 and 7pg/ml, respectively, and no cross-reactivity was observed in detection. All samples were measured in triplicate.

### Statistical analysis

Statistical analyses of data were performed with one-way ANOVA. Correlations were determined by Spearman rank correlation coefficients. Survival was estimated by the Kaplan-Meier method and compared by the log-rank test. The differences between pathological scores were evaluated using the Mann–Whitney *U* test. All data were analyzed with SPSS 17.0. Differences were deemed to be statistically significant at *p*<0.05.

## Abbreviations

AVMC: Acute viral myocarditis; IL-22: Interleukin-22; IL-22R: Interleukin-22 receptor; PBS: Phosphate-buffered saline; HIV: Human immunodeficiency virus; RT-PCR: Real time-polymerase chain reaction; ELISA: Enzyme linked immosorbent assay; IHC: Immunohistochemistry; PFU: Plaque-forming unit.

## Competing interests

The authors declare that they have no competing interests.

## Authors’ contributions

KQ participated in data collection, coordinated the study, performed the statistical analysis and interpretation of data, prepared the draft of manuscript and reviewed it. WW participated in the conception and design, coordinated the study and reviewed it. FY, YL, YX, MG, XP, WL, YY, YP and YD carried out data collection. All authors read and approved the final manuscript.
